# X-linked *ADGRG2* mutation and obstructive azoospermia in a large Pakistani family

**DOI:** 10.1038/s41598-018-34262-5

**Published:** 2018-11-02

**Authors:** Muhammad Jaseem Khan, Nijole Pollock, Huaiyang Jiang, Carlos Castro, Rubina Nazli, Jawad Ahmed, Sulman Basit, Aleksandar Rajkovic, Alexander N. Yatsenko

**Affiliations:** 1 Institute of Basic Medical Sciences, Khyber Medical University, Peshawar, Pakistan; 2 Department of OBGYN and Reproductive Sciences, Magee-Womens Research Institute, School of Medicine, University of Pittsburgh, Pittsburgh, PA, USA; 3 Center for Genetics and Inherited Diseases, Taibah University, Almadina Almunawarrah, Medina, Saudi Arabia; 4 Department of Pathology, University of California San Francisco (current appointment), San Francisco, CA, USA; 5 Department of Pathology, School of Medicine, University of Pittsburgh, Pittsburgh, USA; 6 Department of Genetics, School of Public Health, University of Pittsburgh, Pittsburgh, USA

## Abstract

We performed whole exome sequencing to identify an unknown genetic cause of azoospermia and male infertility in a large Pakistani family. Three infertile males were subjected to semen analysis, hormone testing, testicular histology, ultrasonography, karyotyping, Y-chromosome microdeletion and *CFTR* testing. The clinical testing suggested a diagnosis of obstructive azoospermia (OA). To identify the cause, we performed whole exome sequencing (WES) for 2 infertile brothers and 2 fertile family members. For segregation analysis and variant confirmation, we performed Sanger sequencing. WES data analysis of the family revealed segregated variants in 3 candidate genes. We considered novel nonsense variant c.2440C > T(p.Arg814*) in X-linked gene *ADGRG2* as biologically most plausible. It is predicted to truncate the protein by 204 amino acids (aa) at a key transmembrane domain. *Adgrg2*-knockout male mice show sperm loss due to obstructive fluid stasis, while *ADGRG2* mutations cause OA in the infertile male patients. Our analysis of testicular histology reveals secondary severe reduction of spermatogenesis, consistent with human and knockout mouse phenotypes. The *ADGRG2* nonsense mutation is absent in the largest population databases, ExAC and gnomAD. Analysis of the novel nonsense mutation in extended family members confirmed co-segregation of the mutation with OA in all affected males. The likely pathogenic nature of the mutation is supported by its truncation effect on the transmembrane domain and distinctive ultrasound results. The study demonstrates effectiveness of WES in discovering a genetic cause of azoospermia.

## Introduction

Worldwide, approximately 15% of all couples are affected by infertility, and genetic pathology accounts for up to 50% of male factor infertility^1,2^. It is a major social concern and a source of continuous stress for infertile couples^3,4^. Yet, male infertility is a poorly understood and genetically heterogenous condition. Known genetic forms of infertility affect various reproductive organs, ranging from the pituitary to the gonads^5,6^. Nearly 15% of infertile males suffer from azoospermia in the form of obstructive azoospermia (OA) or non-obstructive spermatogenic failure^7,8^. Differential clinical diagnosis of these forms is based on follicle-stimulating hormone (FSH) level, semen volume, and obstructions in the reproductive tract detected by ultrasonography; often, an elevated level of FSH is detected in non-obstructive azoospermia, while a normal FSH level and low semen volume point to OA^4,9^. At least 50% of azoospermia is estimated to be caused by genetic defects, including numerical chromosome aberrations, *CFTR* mutations, and microdeletions in the azoospermic factor (AZF) regions on chromosome Yq11. Recently with the advent of genomic techniques, a number of genes responsible for non-obstructive azoospermia were identified, *NR5A1 (SF1), HSF2*, *SYCP3*, *SOHLH1*, and *TEX11*^10–14^. However, these established genetic causes still only account for a minority of azoospermic patients.

The majority of OA cases are attributed to congenital bilateral absence of the vas deferens (CBAVD) often caused by *CFTR* mutations^15^. Yet, a significant fraction of OA patients has not been identified as having *CFTR* mutations. Recently, a new X-linked gene responsible for OA, *ADGRG2*, was discovered^16,17^. Targeted deletion of *Adgrg2* in the mouse was reported to cause progressive obstructive infertility due to impairment in the efferent duct fluid reabsorption process^18^. *ADGRG2* encodes adhesion G protein-coupled receptor G2. The receptor belongs to a large family of adhesion-class G protein-coupled receptors (adhesion-GPCRs)^18,19^. Further studies of the mutant gene in mice showed accumulation of fluid in the testis, stasis of spermatozoa within the efferent ducts, and obstructive infertility^20^. Since male infertility is a highly complex and heterogenous disease, it requires an efficient and reliable method to identify the multitude of plausible causative mutations across the genome. One such approach is a whole exome sequencing (WES)^21^. Here we applied WES to identify the cause of OA and male infertility in a large Pakistani family of Pashtun ethnic origin.

## Materials and Methods

### Subjects and clinical examination

This study was approved by the Ethical Review Board of Khyber Medical University, Peshawar, and the Institutional Review Board of the University of Pittsburgh. It was conducted in accordance with guidelines and regulations. An informed consent was obtained from all subjects. The study family members belong to the Khyber Pakhtunkhwa province of Pakistan. Detailed clinical examination was carried out for 3 affected individuals. Semen analysis was performed for III.3, III.5, and III.13 affected males according to WHO 2010 guidelines^22^. Semen analyses were repeated to confirm initial results. Doppler ultrasonography of the scrotum was performed at Rehman Medical Institute, Peshawar. Standard transrectal ultrasonography was performed at Shifa International Hospital, Islamabad. For hormone analysis, blood samples were collected from III.3, III.5, and III.13. Endocrine testing for luteinizing hormone (LH), follicle-stimulating hormone (FSH), testosterone (T), and prolactin (PRL) were analyzed by ARCHITECT chemiluminescent immunoassay (Abbott)^4,23^. For histopathology, testicular biopsies were obtained from patients III.3 and III.5. Tissue was embedded in paraffin blocks, stained with hematoxylin and eosin (H&E), and examined for detailed microscopic structure of the testis^24–26^.

### Clinical genetic testing

Per guidelines of the American Urological Association, patient samples were tested for common genetic causes of azoospermia^4^. Conventional cytogenetic analysis for affected patients III.3 and III.5 was performed^27^. Briefly, peripheral blood samples were collected in heparin tubes (BD Biosciences) and were cultured for 72 hours in RPMI-1640 medium supplemented with fetal bovine serum and phytohemagglutinin. Cytogenetic analysis was performed using the GTG banding technique. At least 30 metaphases were analyzed. Genomic DNA was isolated using the Gentra Puregene blood kit (Qiagen). The concentration of DNA was quantified by the Qubit 2.0 fluorometer (Life Technologies), and purity was analyzed by Nanodrop 2000 spectrophotometer (Thermo Fisher Scientific). Testing for Y-chromosome microdeletions AZFa, AZFb, and AZFc was performed via fragment analysis for PCR amplified regions sY14, sY86, sY127, and sY254 (Suppl. Table 1). The PCR products were analyzed on 1.3% agarose gel^28^. All exons and intron 9 of *CFTR* were reviewed for CBAVD-associated variants from whole exome sequencing data.

### Whole exome sequencing

Two infertile brothers (III.3 and III.5), one fertile healthy brother (III.7), and their mother (II.6) were subjected to whole exome sequencing. For library preparation, SureSelect Human All Exon V6 (Agilent), covering most coding RefSeq genes, was used^29^. Cluster generation and DNA sequencing was performed on a HiSeq. 2500 sequencing machine (Illumina). Briefly, 50 ng of DNA was fragmented using the QXT enzymatic protocol followed by adaptor tagging (Agilent). DNA fragments were purified with magnetic beads, and target regions were captured with whole exome oligonucleotides, followed by amplification of the enriched library. The library was quantified with the Qubit fluorometer and library size distribution was measured with a Bioanalyzer (Agilent). Paired-end sequencing of libraries was conducted using a V4 high-output flow cell (Illumina). The sequencing read length was 125 bp and the read depth coverage was estimated on average >150x per target interval. The raw data were evaluated for read quality with FastQC software (Babraham Bioinformatics). The Burroughs-Wheeler Aligner (BWA), incorporated in BaseSpace with the BWA-MEM algorithm, was used to align FASTQ files to the reference genome. Variants were called using the Genome Analysis Toolkit (GATK, Broad Institute). Genomic variants were annotated with ANNOVAR^30^. Population variant allele frequency was identified in the following databases: ESP6500 (National Heart, Lung, and Blood Institute), 1000 Genomes (NCBI browser), ExAC and gnomAD (The Broad Institute). Variants were evaluated for potential clinical significance using OMIM (Johns Hopkins University), HGMD (Qiagen), and ClinVar (NCBI) databases. Animal models for genes’ variants were evaluated using the MGI database (Jackson Laboratory). Tissue gene expression was evaluated with AceView (National Center for Biotechnology Information, NCBI), BioGPS (Scripps Research Institute), GTEx Portal (Broad Institute), and UniGene (NCBI) databases. Variant conservation was tested with PhyloP (Cornell University) and *Clustal Omega* (EMBL-EBI)^31^. Protein change predictions were done by *PolyPhen-2* (Harvard University), *SIFT* (J. Craig Venter Institute), and *MutationTaster* (Charité). Sanger sequencing confirmation of WES variants was performed with BigDye sequencing kit (ThermoFisher Scientific). PCR primers were designed using *Primer3*+ (Suppl. Table 1)^32^. Sanger sequence analysis was performed with Sequencher (GeneCodes).

## Results

### Family history and clinical analysis

**Figure 1:**
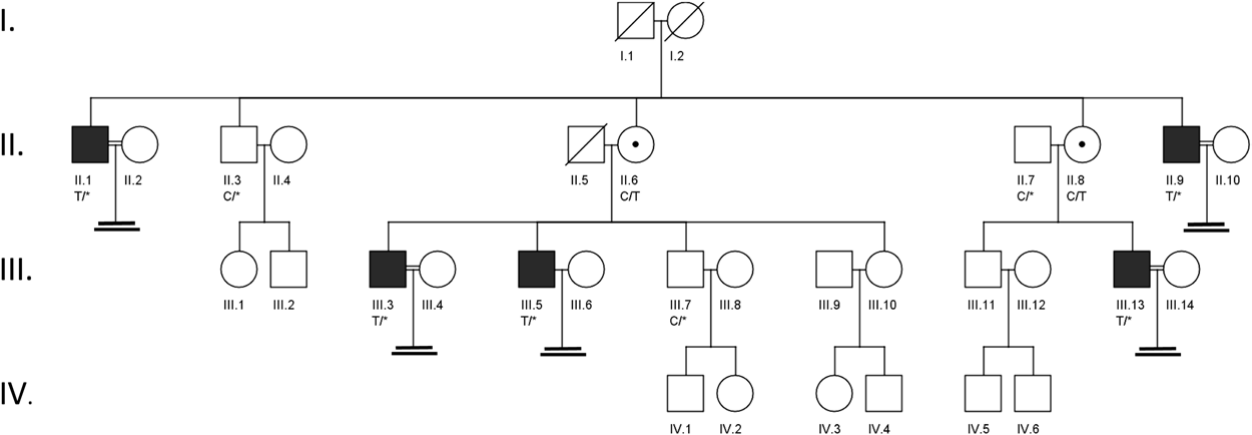
(**A**) Pedigree of a family with OA and male infertility. Members III.3, III.5, and III.13 have male infertility diagnosed with azoospermia. Affected males are shown as black rectangles, females as white circles, and female carrier as a circle with a dot in the center. Corresponding C/T genotype in *ADGRG2* variant c.2440C > T (p.Arg814*) is shown below each tested female family member. A hemizygous variant “T/*” is shown in males due to its absence on the Y chromosome. Based on segregation analysis, family member I.2 was the earliest known female carrier of the *ADGRG2* nonsense variant. The mutation may have originated in the germline of the member I.2, or it may have been inherited from previous generations through the female line. Consanguineous relationships are shown with a double horizontal line. While consanguinity was noted, the consanguineous couples did not produce offspring and did not affect the genetic analysis. DNA samples from II.6, III.3, III.5, and III.7 were studied with WES.

**Figure 2:**
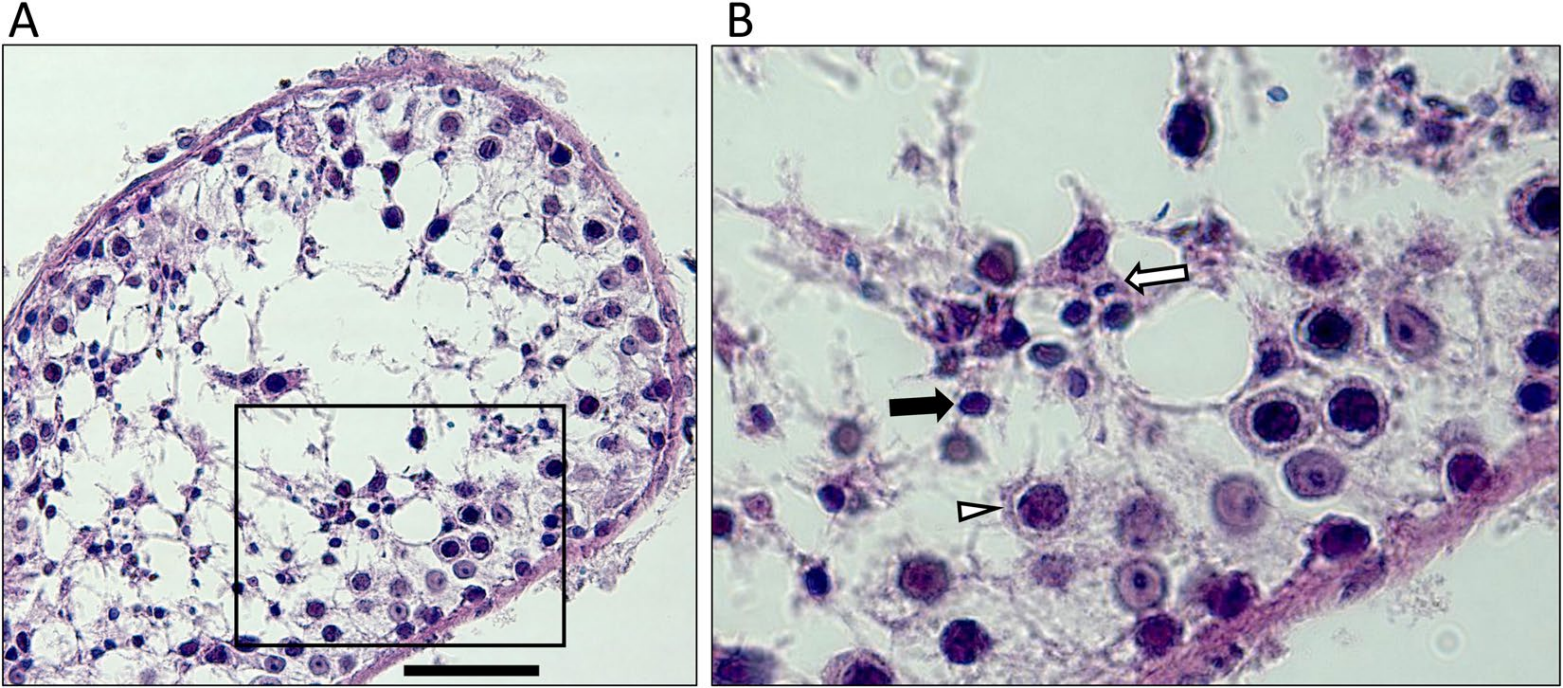
Histology of testicular tissue of azoospermic male III.3. Formalin-fixed paraffin-embedded tissue was stained with hematoxylin-eosin. Scale bar indicates 50 µm. Severe secondary hypospermatogenesis and spermatocyte stage arrest were observed. Emptied areas filled with building fluid are dispersed inside the tubule. Most cells identified are germ cells from earlier stages, such as spermatocytes (white triangle), and round spermatids (black arrow), with a few elongating spermatids (white arrow).

A large four-generation Pakistani family which included five infertile males was studied (Fig. 1A). Semen analysis of three males with primary infertility, III.3, III.5, and III.13, revealed absence of sperm and reduced semen volume of 0.9, 1.0, and 1.2 ml, respectively (Table 1). Samples showed normal semen viscosity, normal liquefaction time, and acidic pH in III.3 and III.5. Semen analysis in proband III.3 showed presence of fructose in semen, indicating that the ejaculatory duct and connected seminal vesicle were not completely occluded. Hormone testing was unremarkable, indicating no hormonal cause of infertility (Table 1). Testosterone levels were slightly lower in affected males and FSH, luteinizing hormone (LH) and prolactin (PRL) were consistently normal. None of the patients had symptoms of classic cystic fibrosis. Scrotal ultrasonography in male III.3 detected present vas deferens, normal blood supply, and normal testicular volume, normal epididymi without nodulation nor calcification, and no cystic lesions. Mild bilateral hydrocele and multiple dilated tortuous blood vessels in the pampiniform plexus of ~2.3 mm was noted in left scrotal sac. Scrotal ultrasonography of male III.13 showed testicular hypotrophy for right (1.3 × 1.6 cm) and left (1.2 × 1.5 cm) sides. Transrectal ultrasound (TRUS) testing of proband III.3 indicated bilateral absence of the vas deferens, an absent right seminal vesicle, and the left seminal vesicle reduced in size with a 5 mm diameter. No inflammation or lesions were detected, and the prostate was of normal size at 21 g. The testis histology of proband III.3 showed dilated seminiferous tubules with reduced spermatogenesis (Fig. 2). A moderate edema of interstitial tissue and mild hyperplasia of Leydig cells was also seen. Cell differentiation stages from spermatogonia to elongated spermatids were observed with a low number of spermatids and spermatozoa. Microscopic examination in patient III.5 revealed a similar phenotype (data not shown); seminiferous tubules were separated by delicate fibrovascular interstitium with clusters of Leydig cells. The tubules were lined by Sertoli cells, primary and secondary spermatogonia, and rare spermatids. Conventional cytogenetic analysis in these patients revealed normal 46,XY male karyotypes. AZF deletion testing for all affected males was negative. Sequence analysis of all *CFTR* exons and intron 9 did not identify pathogenic CBAVD-associated mutations (Suppl. Table 3). Two rare *CFTR* likely benign variants were identified in family members, but they did not segregate with the male infertility phenotype.

**Table 1:** Summary of clinical laboratory evaluation for infertile males.

ID#	Age	Sperm count	Semen volume (mL)	pH	T*, (3–12 ng/mL)	PRL**, (units vary)	FSH, (1.5–12.4 IU/L)	LH, (1.8–8.6 IU/L)	Scrotal ultrasound testing	Karyotype	AZFs del	*ADGRG2* Mutation
III.3	42	0	0.9	7.0	1.9	10 ng/mL (0–20 ng/mL)	4.3	3.1	bilateral mild hydro cele, left varicocele, 2.3 × 3.1 cm (right), 2.1 × 3 cm (left)	Normal 46, XY male	Norm	c.2440C > T
III.5	29	0	1	7.0	2.1	NA	6.5	5.8	NA	NA	Norm	c.2440C > T
III.13	35	0	1.2	8.0	2.95	162 IU/L (0–425 uIU/mL)	9.07	7.55	Hypotrophy, 1.3 × 1.6 cm (right), 1.2 × 1.3 cm (left)	Normal 46, XY male	Norm	c.2440C > T

Semen analysis of III.3, III.5, and III.13 males indicated low semen volume (<1.5 mL) and slightly acidic semen pH (<7.2) in III.3 and III.5 males, both characteristic of obstructive azoospermia. Due to absent sperm, other testing, such as motility and morphology, was not performed. Abnormal values are indicated in bold. T: testosterone, PRL: prolactin, FSH: follicle stimulating hormone, LH: luteinizing hormone, AZFs: Y chromosome regions AZFa, AZFb, and AZFc. Results indicated as “NA” were not collected. “Norm” designates normal findings. T*- Testosterone levels were collected outside of the recommended timeframe (7am-10am). PRL** - different units used by different laboratories.

### Whole exome sequencing mutation analysis

Whole exome sequencing (WES) of two affected brothers (III.3 and III.5), one fertile brother (III.7), and the mother (II.6) produced ~400 million raw reads, with ~380 million high-quality reads aligned to the GRCh38/hg38 reference genome (Suppl. Table 2). Overall, a total of 50,988 high-quality variants were identified (Suppl. Fig. 1). Variants in coding exon and flanking intronic regions (within 10 bp) accounted for 29,638 variants, while 12,587 were nonsynonymous (Suppl. Table 3). These variants were reduced to 1,530 having a minor allele frequency (MAF) of <1% in the 1000 Genomes, EVS6500, ExAC, and gnomAD genome databases. Among these rare coding nonsynonymous variants, 171 showed genotype co-segregation with OA in the pedigree, namely both affected brothers showed the same genotype, while the unaffected brother had a different genotype. Predicted loss of function variants (LoF) from co-segregating variants (n = 171) included nonsense (n = 3), frameshift (n = 9), splicing (n = 1), and non-frameshift insertion/deletions(n = 12), (total n = 25) (Suppl. Fig. 1 and Suppl. Table 3). All co-segregating variants were prioritized based on respective gene tissue expression, biological (MGI), clinical (OMIM, ClinVar), and inheritance information. We considered X-linked and recessive models to be of highest priority. Thereby, we identified two hemizygous variants and one autosomal homozygous variant in three genes - *ADGRG2*, *P2RY4*, and *CLEC6A*, respectively (Table 2). Genes *P2RY4* and *CLEC6A* were ruled out due to absence of infertility phenotype in a mouse model (MGI), nonspecific expression in testis tissue in 4 expression databases (AceView, BioGPS, GTEx, UniGene), and lack of evidence for genetic variants linked to infertility in clinical cases (OMIM and ClinVar databases). Aside from *ADRGR2* and *PIWIL3*, none of the LoF variants had a predicted likely role in male fertility. The single heterozygous variant in *PIWIL3* was ruled out because it is insufficient for autosomal recessive inheritance.

**Table 2:** Whole exome sequencing top 3 candidate variants.

Variant #	Chr	Gene, NCBI #	DNA change	Protein change	SNV type	Geno type	Knockout mouse model	ClinVar	Testis gene expression	PhyloP	SIFT	PolyPhen2
1	X	*ADGRG2* NM_001079858	c.2440C > T	p.Arg814*	Nonsense	Hemizygous	Male infertility, luminal fluid reabsorption defect	5 pathogenic CBAVD mutations	Epididymis, not test.specific	C	NA	NA
2	X	*P2RY4* NM_002565	c.59G > A	p.Ser20Asn	Missense	Hemizygous	Lack of chloride secretion in GI tract, fertile	Not reported	Not testis-specific	N	T	B
3	12	*CLEC6A* NM_001007033	c.23A > C	p.Gln8Pro	Missense	Homozygous	Low cytokine production & immune response to fungi	Not reported	Not testis-specific	N	T	D

The variants were identified as the most plausible for male infertility. Selection was based on inheritance pattern, genotype segregation with OA, gene and variant priority. Mouse models were retrieved from MGI database. Testis gene expression is based on consistent results from 4 databases: BioGPS, GTEx, AceView, and UniGene. C: Conserved, N: Not conserved, T: tolerated, B: benign, NA: not available.

**Figure 3:**
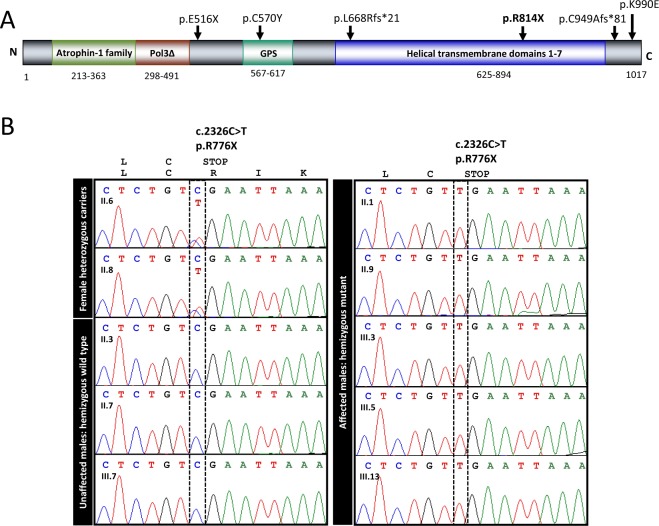
(**A**) Map of the identified *ADGRG2* variant and protein domains. The novel nonsense variant p.Arg814* in *ADGRG2* is shown in bold. It is mapped to the second half of transmembrane 7-helical domain. Previously reported pathogenic variants in patients with OA are indicated above the protein in plain. Predicted domain consensus positions shown below. The extracellular region Atrophin-1 & Pol3∆ denotes two overlapping regions, Atrophin-1 (p.213–363) is showing homology to Atrophin-1 superfamily and Pol3∆ is DNA polymerase III, delta subunit superfamily (p.298–491), also known as HolA. GPS denotes GPCR proteolytic site motif. They were predicted using conserved domains, CDD/SPARCLE (NCBI), UniProt, and InterPro. (**B**) Sanger sequencing confirmation of *ADGRG2* mutation in family members. The novel nonsense variant in *ADGRG2* was confirmed through Sanger sequencing. The variant co-segregated with OA in all family members sequenced: all affected males carry the mutant allele, all unaffected males carry the wild type allele, and both mothers of affected sons carry one copy of the mutant allele.

The remaining variant was a nonsense change in *ADGRG2*, an X-linked gene that encodes adhesion G protein-coupled receptor G2. Human ADGRG2 protein is expressed primarily in the epididymis and efferent ducts, but not in the seminiferous tubules^16,33^. Null *Adgrg2* mutations in male mice cause obstructive azoospermia due to fluid buildup in testis and spermatozoa stasis in efferent ducts^18^. Notably, three loss-of-function mutations in the *ADGRG2* gene were reported in infertile males with OA (Fig. 3A). Our identified *ADGRG2* variant c.2440C > T(p.Arg814*) introduces a premature stop codon that truncates the protein by 204 aa and disrupts a highly conserved transmembrane domain (Fig. 3A). This variant is absent in databases of genetic variants of the general population, suggesting its high pathogenicity^34^. The amino acid position p.Arg814 is conserved in all vertebrate taxa known to have the gene. Most of the ADGRG2 helical transmembrane domain surrounding the variant is highly conserved as well (Suppl. Fig. 2). Following segregation analysis of the mutation in all available family members confirmed co-segregation of the *ADGRG2* variant with OA in all infertile males (Fig. 3B).

## Discussion

Obstructive azoospermia and congenital bilateral absence of the vas deferens (CBAVD) were long considered a single-gene disorder. *CFTR* mutations result in pathology of the genitourinary system with complete failure to develop a channel transporting sperm from testis to urethra. The gene mutations cause nearly 80% of the disease load^35^. While the *CFTR* contribution is the most common cause reported to date, there are still many infertile patients with idiopathic OA who have no identified genetic cause. A recent study of OA patients with CBAVD and *CFTR*-negative results reported loss-of-function mutations in *ADGRG2* in 3/26 patients (~12%). Another study in a Chinese population identified missense mutations in a small fraction of OA patients, suggesting varied incidence of *ADGRG2* mutations in different ethnic groups^17^. While truncating mutations were reported in the gene previously^16,17^, here we reported the first Pakistani family with an *ADGRG2* mutation. This study offers strong evidence with mutation co-segregating with phenotype in 5 affected males and 5 additional family members.

The affected men in this family were diagnosed with OA based on ultrasound findings, low semen volume, no sperm in ejaculate, and normal hormone levels. Clinical semen analysis in affected siblings suggested obstructive azoospermia, presenting with reduced semen volume, ranging from 0.9 ml to 1.2 ml. Ejaculatory duct obstruction was unlikely because patient III.3 had positive semen fructose, indicating some contribution to the ejaculate from the left seminal vesicle^4^. TRUS results also supported this finding, showing none of the characteristic signs of ejaculatory duct obstruction such as dilated seminal vesicles, inflammation, or lesions. Scrotal ultrasound testing indicated present vas deferens. However, TRUS testing in available affected patients indicated incomplete bilateral absence of the vas deferens, and unilateral absence of the right seminal vesicle. Such incomplete bilateral absence of the vas deferens is consistent with previous reports^16^.

*ADGRG2* is expressed in the epididymis and the efferent ducts, yet adhesion G protein-coupled receptor G2 is critical for normal function of the efferent ducts in the testis^36^. The ducts reabsorb fluid, which carries immature spermatozoa. Null male mice show a disrupted reabsorption process that leads to the accumulation of fluid in the ducts, suggesting that the protein regulates fluid reabsorption. Mutant hemizygous males presented with dilated efferent ducts, with a large number of spermatozoa accumulated in the lumen of the ducts, dilated seminiferous tubules with hypospermatogenesis, and severely reduced fertility that progresses to complete infertility with age. A similar phenotype was found in some patients with loss of function in ADGRG2, who showed enlarged sections of the epididymal head and some dilated efferent tubules in histological studies^16^. Since the efferent ducts are responsible for over 90% of fluid reabsorption, over time it leads to dilated seminiferous tubules with a reduced number of sperm. Similarly, the observed hypospermatogenesis in the proband III.3 is likely a secondary effect due to ADGRG2 protein loss of function, defective fluid reabsorption in the efferent ducts and a retrograde effect of fluid on the seminiferous tubules^18^. This is also supported by ADGRG2 tissue expression profile, where human ADGRG2 protein is expressed primarily in the epididymis and efferent ducts, but not in the seminiferous tubules^16,33^. Since the protein is not expressed in the seminiferous tubules, it cannot disrupt spermatogenesis directly.

Previous studies of *ADGRG2*-positive patients with obstructive azoospermia have been limited to histology of the epididymis, and here we show the first examples of hypospermatogenesis identified from testicular biopsy in two patients with an *ADGRG2* nonsense mutation. Patients with CBAVD typically have normal spermatogenesis, although *CFTR* negative CBAVD patients with unexplained testicular hypospermatogenesis have been described^37^. Here, proband III.3 had mildly reduced testis size, while patient III.13 had more severe hypotrophy, likely reflecting phenotypic variability^38^. The degree of testicular hypotrophy in the proband III.3 was comparable to *Adgrg2* null mouse model phenotype where mice showed progressive hypospermatogenesis with age^18^. Complete atrophy was not detected, and mature sperm were still found in testis of aged mice. OA phenotype caused by *ADGRG2* mutations is thus different from CBAVD caused by *CFTR* mutations and may affect different components of the development of the urogenital tract. Mouse model evidence further supports this difference, mice with *CFTR* loss of function often have normal male fertility^39,40^, while *Adgrg2* null mice have a phenotype more comparable to our clinical findings in humans. Yet, more studies are needed to identify the role of ADGRG2 protein.

Thus, our collective evidence from clinical testing, including semen analysis, endocrinology, ultrasonography, histopathology, and genetics, coupled with segregation analysis of family members, presents convincing evidence for the *ADGRG2* mutation as a cause of obstructive azoospermia and offers new insight into how ADGRG2 may cause OA.

## Electronic supplementary material

Supplementary Figures

Supplementary Dataset 1,2,3,4,5,6
