# Supplementary material for: X-linked ADGRG2 mutation and obstructive azoospermia in a large Pakistani family
Source: Sci Rep. 2018 Nov 2;8:16280. doi: 10.1038/s41598-018-34262-5 (PMC6214919; doi:10.1038/s41598-018-34262-5)
Supplement: Supplementary file 1 — Supplementary Figures [file 41598_2018_34262_MOESM1_ESM.docx]

**X-linked *ADGRG2* mutation and obstructive azoospermia in a large Pakistani family**

**Muhammad Jaseem Khan^1^, Nijole Pollock^2^, Huaiyang Jiang^2^, Carlos Castro^2^, Rubina Nazli^1^, Jawad Ahmed^1^, Sulman Basit^3^, Aleksandar Rajkovic^2, 4^, Alexander N. Yatsenko^2^**

^1^Institute of Basic Medical Sciences, Khyber Medical University, Peshawar, Pakistan

^2^Department of OBGYN and Reproductive Sciences, Magee-Womens Research Institute,

University of Pittsburgh

^3^Center for Genetics and Inherited Diseases, Taibah University, Almadina Almunawarrah, Saudi Arabia

^4^University of California San Francisco, Department of Pathology (current appointment)

**Supplemental Figure 1.**

Total variants (n=54,527)

GATK quality variants (n=50,988)

Coding exome variants (n=29,638)

Nonsynonymous variants (n=12,587)

Minor allele frequency <1% (n=1,530)

Genotype co-segregating with OA (n=171, LoF=25, M=146)

Homozygous (n=1, LoF=0, M=1)

Hemizygous (n=2, LoF=1, M=1)

Multiple heterozygous (n=27, LoF=10, M=17)

Single heterozygous (n=141, LoF=14, M=127)

Gene and variant review: **3 variants in 3 genes**

**Supplemental Figure 1. Whole exome sequencing data process and analysis.** The anticipated inheritance pattern was an identical genotype in 2 affected brothers and a different genotype in the unaffected brother (n=171). The co-segregating variants were categorized as either potentially loss of function (LoF, n=25) or missense (M, n=146). The LoF variants included nonsense, frameshift, splicing, and non-frameshift insertion/deletion variants. The variant genes were reviewed for mouse model phenotypes, clinical findings, and testis-specific gene expression as described in the text. The variants with autosomal recessive (n=1) and X-linked (n=2) inheritance patterns were considered most likely to be significant and were reviewed in depth.

**Supplemental Figure 2.**

*Homo sapiens* FEKIRRDYPSKILIQLCAALLLLNLVFLLDSWIALYK-MQGLCISVAVFLHYFLLVSFTW 711

*Pan troglodytes* FEKIRRDYPSKILIQLCAALLLLNLVFLLDSWIALYK-MQGLCISVAVFLHYFLLVSFTW 749

*Mus musculus* FEKIRRDYPSKILIQLCAALLLLNLIFLLDSWIALYN-TRGFCIAVAVFLHYFLLVSFTW 703

*Rattus norvegicus* FEKIRRDYPSKILIQLCAALLLLNLVFLLDSWIALYN-ARGFCISVAVFLHYFLLVSFTW 707

*Equus caballus* FEKIRRDYPSKILIQLCAALLLLNLVFLLDSWIALYD-MRGLCISVAVFLHYFLLVSFTW 712

*Gallus gallus* FEKIRRDYPSKILIQLCAALLLLNLVFLLDSWIALYN-TRGLCIAVAVFLHYFLLVSFTW 1041

*Xenopus tropicalis* FEKIRRDYPSKILMQLCAALILLNLTFLINPWIALYNNIPGLCISAAAFLHYFLLVSITW 338

*Takifugu rubripes* FEKLLRDIPAKILVQLCVSLLLLNLLFLLDGWLAQHP-SSGLCISTAFFLHYFLLTSFTW 994

***: ** *:***:***.:*:**** **:: *:* : *:**:.* *******.*:**

*Homo sapiens* MGLEAFHMYLALVKVFNTYIRKYILKFCIVGWGVPAVVVTIILTISPDNYGLGSYGKFPN 771

*Pan troglodytes* MGLEAFHMYLALVKVFNTYIRKYILKFCIVGWGVPAVVVTIILTISPDNYGLGSYGKFPN 809

*Mus musculus* MGLEAFHMYLALVKVFNTYIRKYILKFCIVGWGIPAVVVSIVLTISPDNYGIGSYGKFPN 763

*Rattus norvegicus* MGLEAFHMYLALVKVFNTYIRKYILKFCIVGWGIPAVVVSIVLTISPDNYGIGSYGKFPN 767

*Equus caballus* MGLEAFHMYLALVKVFNTYIRKYILKFCIVGWGVPAVVVTIVLIISPDNYGLGSYGKFPN 772

*Gallus gallus* MGLEAFHMYLALVKVFNTYVRKYILKFCIVGWGLPAVVVAIVLAVSPDNYGLISTGRVSK 1101

*Xenopus tropicalis* MGLEAFHMYFSLVKVFNTYVRKYILKFCIVGWGVPAVVVAIILAVNKDLYGFQSKGKYPN 398

*Takifugu rubripes* AGLEALHMYLSVVQVFLPYLSRYMLKVSLIGWGLPLLVVIVTISVDKDNYGLVPYSKHSD 1054

****:***:::*:** *: :*:**..::***:* :** : : :. * **: .: .

p.Arg814*

*Homo sapiens* GSPDDFCWINNNAVFYITVVGYFCVIFLLNVSMFIVVLVQLC**R**IKKKKQLGA-QRKTSIQ 830

*Pan troglodytes* GSPDDFCWINNNAVFYITVVGYFCVIFLLNVSMFIVVLVQLC**R**IKKKKQLGA-QRKTSIQ 868

*Mus musculus* GTPDDFCWINSNVVFYITVVGYFCVIFLLNVSMFIVVLVQLC**R**IKKKKQLGA-QRKTSIQ 822

*Rattus norvegicus* GTPDDFCWINSSVVFYITVVGYFCVIFLLNVSMFIVVLVQLC**R**IKKKKQLGA-QRKTSIQ 826

*Equus caballus* GSPDDFCWINSNAVFYITVVGYFCVIFLLNVSMFIVVLVQLC**R**IKKKKQLGA-QRKTSIQ 831

*Gallus gallus* TRPDEFCWIKNRIVFYITAVGYFCVIFLINISMFIVVLIQLC**R**IKKKKQLGA-QRKTSIQ 1160

*Xenopus tropicalis* GDSDDICWIA-DIIFYITVVGYYGIVFLMTISMFIVVILQLC**R**IKKQKQLGF-QKKITLQ 456

*Takifugu rubripes* GTSDKFCWLRNDVAFYVGVVAYFLLVFALCLLVFIMVMVQLA**R**IKRQNPHNQSPSRGVLT 1114

*.:**: **: .*.*: ::* : : :**:*::**.***::: . : :

*Homo sapiens* DLRSIAGLTFLLGITWGFAFFAWGPVNVTFMYLFAIFNTLQGFFIFIFYCVAKENVRKQW 890

*Pan troglodytes* DLRSIAGLTFLLGITWGFAFFAWGPVNVTFMYLFAIFNTLQGFFIFIFYCVAKENVRKQW 928

*Mus musculus* DLRSIAGLTFLLGITWGFAFFAWGPVNVTFMYLFAIFNTLQGFFIFIFYCAAKENVRKQW 882

*Rattus norvegicus* DLRSIAGLTFLLGITWGFAFFAWGPVNLTFMYLFAIFNTLQGFFIFIFYCAAKENVRKQW 886

*Equus caballus* DLRSVAGLTFLLGITWGFAFFAWGPVNVTFMYLFAIFNTLQGFFIFIFYCVAKENVRKQW 891

*Gallus gallus* DLRSVAGLTFLLGITWGFAFFT---VNEVFTYLFTIFNTLQGFFIFIFYCVTKENVRKQW 1217

*Xenopus tropicalis* DMRSVAGITFLLGITWGLAFFSWGPGGVVIVYLFTIFNTLQGFFIFIFYCVAKENVRKQW 516

*Takifugu rubripes* DVRSITGLVILLGLTWGFALFSWGPLVLPFTYLFSIFNSLQGFLVFVFHCAAKENVRRQW 1174

*:**::*:.:***:***:*:*: : ***:***:****::*:*:*.:*****:**

**Supplemental Figure 2. Conservation of the ADGRG2 helical transmembrane domain in vertebrates.** Amino acid residues of the helical transmembrane domain, indicated in the red box, are conserved among vertebrates. The variant p.Arg814*, indicated in bold, is highly conserved. Below the alignment, positions with consistently identical residues are indicated by asterisk, ”*”. Based on the Gonnet PAM 250 [[41](#_ENREF_41)] matrix positions with similar categories of amino acid were indicated with a colon “:”, or weakly similar (score =< 0.5) with a period “.”, and blank space indicates no predicted conservation. Alignment was generated by Clustal Omega Multiple Sequence Alignment (MSA). Transcripts used are: *Homo sapiens*: NP_001073327.1, *Pan troglodytes*: XP_016798679.1, *Mus musculus*: NP_848827.1, *Rattus norvegicus*: NP_852031.1, *Equus caballus*: XP_005614086.1, *Gallus gallus*: XP_025002348.1, *Xenopus tropicalis*: XP_017946974.1, *Takifugu rubripes*: XP_011606416.1

**Supplemental Table 1.**

| Region | Primer name | Product size (bp) | Sequence |
| --- | --- | --- | --- |
| AZFa | sY86-F | 326 | 5'-GTG ACA CAC AGA CTA TGC TTC-3' |
|  | sY86-R |  | 5'-ACA CAC AGA GGG ACA ACC CT-3' |
| AZFb | sY127-F | 274 | 5'-GGC TCA CAA ACG AAA AGA AA-3' |
|  | sY127-R |  | 5'-CTG CAG GCA GTA ATA AGG GA-3' |
| AZFc | sY254-F | 380 | 5'-GGG TGT TAC CAG AAG GCA AA-3' |
|  | sY254-R |  | 5'-GAA CCG TAT CTA CCA AAG CAG C-3' |
| ADGRG2 | ADGRG2 ex23-F | 684 | 5'-TGG CAA ACA GAT ACA TGA AGG TC-3' |
|  | ADGRG2 ex23-R |  | 5'-CTG GAA ATG CCA TGC TAC TCT TC-3' |

**Supplemental Table 1. PCR primers for Y chromosome microdeletions testing and *ADGRG2* sequencing.** DNA sequence for forward and reverse primers shown for the regions AZFa, AZFb, and AZFc. Testing was performed with primers recommended by the 2013 EAA/EMQN best practice guidelines. All samples yielded a strong visible PCR product of the correct size. Gel analysis ruled out common AZFa, -b, and -c microdeletions. *ADGRG2* PCR primers were used for Sanger sequencing as well.

**Supplemental Table 2.**

| Sample | Total aligned reads | Total high quality aligned reads | Q20 bases | Q20 bases % | Target Q20 bases | Target Q20 bases % | Target coverage | exons >10x coverage |
| --- | --- | --- | --- | --- | --- | --- | --- | --- |
| II.6 | 113188687 | 108866197 | 13175147243 | 96% | 10443275320 | 79% | **172x** | 98.90% |
| III.3 | 105273066 | 101032179 | 12229958034 | 96% | 9662433033 | 79% | **159x** | 98.70% |
| III.5 | 84684265 | 81309104 | 9884111301 | 97% | 7840058819 | 79% | **129x** | 98.40% |
| III.7 | 93613615 | 89788970 | 10808112159 | 96% | 8573378153 | 79% | **141x** | 98.60% |

**Supplemental Table 2. Whole exome sequencing quality and coverage.** Individual reads quality was initially evaluated for quality using FastQC software (Babraham Bioinformatics). Reads were aligned to hg19 reference with the Burrows-Wheeler Aligner BWA-MEM algorithm. Individual bases quality was evaluated using Phred score Q20. If the score was >20, it indicates probability of an incorrect base call of 1%. Target findings were based on sequence re-alignment by GATK (Broad Institute) to the reference library of Agilent Human All Exon V6 (Agilent). Target coverage was the average number of reads aligned to any given base at target region. The minimum acceptable quality standard for variant calls was 10x coverage, with >98% of all exons sequenced meeting this criterion in all family members.

**Supplemental Table 3. Whole exome sequencing data.** Initial steps of the WES data bioinformatic analysis are shown in the supplementary data file. Tab 1 contains sorted nonsynonymous variants, tab 2 contains variants with minor allele frequency <1%, tab 3 lists variants that co-segregate with OA, tab 4 contains homo- and hemizygous variants, tab 5 has multiple heterozygous, and tab 6 contains single heterozygous variants.
